# 

**Published:** 1889-10-05

**Authors:** 


### The Hospital

A Weekly Institutional Journal of

### Science, Medicine, Nursing, and Philanthropy.

EDITED BY

HENRY C. BURDETT,

AUTHOR OF "HOSPITALS AND THE STATE"; "PAY HOSPITALS OF THE WORLD"; "COTTAGE HOSPITALS, GENERAL, FEVER, AND CONVALESCENT, WITH FIFTY BEDS AND UNDER: THEIR ORGANISATION, MANAGEMENT, AND WORK"; "BURDETT'S HOSPITAL ANNUAL" "HELP IN SICKNESS" "HELPS TO HEALTH"; ETC., ETC.

ACTING EDITOR:

GEORGE W. POTTER, M.D.,

AUTHOR OF PAPERS ON "BACTERIOLOGY"; "SCARLET-FEVER"; "RHEUMATISM"; ETC., ETC.

SURGEON GEN'L'S OFFICE

130675

LIBRARY

INDEX TO VOL. VII.

(5th October, 1889, to 29th March, 1890.)

FOR 1889–90.

LONDON:

PUBLISHED BY "THE HOSPITAL" (Limited), 140, STRAND, W.C.

W1
H069X

### INDEX TO VOL. VII. 1889-90.

Abraham, Dr. P. S., on the Etiology of Leprosy, 187
Abroad, Going, 182
Absinthe, 155
Abuse, Birmingham Scheme for Inquiring into Hospital, 404
——— of Hospitals, The, 150, 243
——————— at Birmingham, Conference Concerning the, 262, 278
——— of Medical Charity, Birmingham Doctors on the, 121
Accidents (Street) at Liverpool during Twelve Months, 137
Accounts, The Charity Organization Society on Hospital, 405
Acetanilide, 299, 394
Adams, Death of James E., 311
Addenbrooke's, Proposed Ward for Sick Nurses at, 263
Adulteration of Drugs, 315
——————— Food, Some Unpleasant Truths about, 133, 184.
Africa, Human Development in, 248
Ague, Treatment of, 282
Ainhum, 27
Air into the Circulation, The Effect of the Entrance of, 205
Albuminuria in Pregnancy, 107
Alcohol in Medicine, 107
——————— Hospitals, 360
Alcoholic Paralysis, 188
Amaurosis, Hysterical, 249
Ambulance Carriage at the Beckett Hospital, 112
——————— Pupils at the Polytechnic, 153
——————— Service for London, A Street, 168
Ambulances, Epidemics and, 193
American Charities, 88
——————— Public Health Association, Meeting of the, 8, 9
Anæmia, Pernicious, 107, 313
Anæsthesia, Obstructed Breathing from, 283
Anatomical Act, The Paddington Board of Guardians on the, 137
Andeer on the Prevention of Vomiting, 364
Aneurism, Aortic, 11, 281
——————— Traumatic Subclavian, 12
Antifebrin, 13, 92
Antig, Dr., on Treatment of Anæmia, 156
Antimony, Use of, 347
Antipyrin, 12, 60, 92, 234, 250
Antiseptic Dressing, Lister's New, 142
——————— Dressings, 174, 235
——————— Ventilation for Hospitals and Sanatoriums, 68
Antiseptics, 44
——————— Dr. Kendal Franks on, 105
——————— v. Plain Water, 232
Anti-vivisection Hospital, An, 208
Anti-vivisectionists, 25, 73
Appeals and Charities, 97
Archangelsky, Dr., on Antipyrin, 234
Arlidge, Dr., on the Causes of Diseases in Manufacturing Pursuits, 40
Arsenic in Spongy Bone, Accumulation of, 234
Asphalte and Horse Accidents, 186
Asthma, Dr. Dieulafoy on the Treatment of, 408
——— Infirmary for 100 Beds, A Plan for an, 110, 111
——— Reports, Summary of, 4
Asylums Board Ambulances and Epidemics, The Metropolitan, 168, 193
——————— Reports of the Metropolitan, 247
Atropine, 109

Bailey, Mr. H. F., on Albuminuria in Pregnancy, 107
Balfour of Burleigh, Lord, on Paying Patients at the London Fever Hospital, 169
Ballard, Dr., on Antiseptic After-treatment of Vaccination, 408
Banstead Asylum, 262
Bantock, Dr. G. Granville, on Ovariotomy and Listerism, 265
Barker, Mr. A. J., on Intestinal Strangulation, 314
Barmaids and their Friends, 392
Barnet Cottage Hospital, First Report of the, 278
Bashire, Dr. Harvey D., on False Croup, 800
Batten, Dr., and Mr. Bokenham on Antipyrin, 12
Belladonna for Removing Renal Calculi, Toxic Doses of, 188
Bennett, Mr. W. H., on Varicose Veins, 12
——— Sir J. Risdon, on Chest Diseases, 345
Berry, Mr. James, on Goitre, 345
Besant, Mr. Walter, The Latest Morality of, 196
Besnier, Mr. E., on Iodides for Scrofulous Children, 156
Birmingham and Hospital Abuse, 262, 278
——————— Midland Hospital for Women, Unsatisfactory Report of the, 405
——————— the Infectious Diseases Act, 17
——————— General Hospital, Appeal of, 10
——————————— Description of, 54
——————— Hospital Sunday Fund, Amount of the, 105
——————— Scheme for Inquiring into Hospital Abuse, 404
——————— The District Nursing Society at, 101
——————— General Dispensary at, 126
——————— Jaffray Suburban Hospital at, 79
——————— Queen's Hospital at, 40, 94
Bleeding, Dr. Knott on, 75
Blyth, Mr. Wynter, on Life-Seeds and Disinfection, 271
Boeckel, Dr., on Antisepsis, 235
Bollinger, Dr., on Obesity, 11
Belton Woods Board School, Alarming Effect of Bad Smells at, 154
Bombay, Gift towards Founding a Leper Hospital at, 342
——— Medical and Physical Society on Leprosy, The, 139
Bond, Dr. F. F., on Hepatic Abscess, 249
Bookkeeping and Accounts, Hospital, 375
Boro-glyceride as a Skin Disinfectant in Scarlet Fever, 14
Bournemouth, Among the Pines at, 148
——————— as a Health Resort, 333
——————— Royal Victoria Hospital, Report of the, 262
Bowie, Dr. A., on the Inhalatory Treatment of Phthisis, 59
Boyd, Mr. R. W., on Sanitation, 137
Bradford Joint Hospital Fund, 311
——————— Workhouse, 88
Brain, Tumour of, and Anti-Vivisectionists, 25
——— Wounds of the, 141
Bramwell, Mr. Byrom, on Exophthalmic Goitre, 393
Brighton, Sussex County Hospital, Alleged Mismanagement at, 367
British Medical Association on Board School Children, 201
——————————— Dr. Rentoul's Scheme, The South London Branch of the, 127
——— Nurses' Association and the Medical Council, The, 180
Broadwood, Miss, on Cottage Nurses, 268
Bromide of Potassium, Accumulation in the Organism of, 157
Brompton Consumption Hospital, 153
Brothers, Dr. A., on Croup, 345
Brown, Dr. Charles W., on Surgical Dressings, 188
Browning and Vivisection, Mr., 186
Browning's Views of Life and Death, Robert, 260
Bruce, Dr. J. Mitchell, on Difficult Diagnosis, 393
Brunton, Dr. Lauder, on Microbes, 27, 75
Bryant, Dr. Joseph D., on Hernia, 378
——— Mr., on Colotomy, 204
Buchman, Dr., on Typhoid Fevers, 107
Burdett, Mr. Henry C., on Helping the London Poor, 101
——————————— the Hospital Problem, 353, 370, 385
Burial, Earth-to-Earth, 38
Butchers' Shops, 9
Buzzard, Dr., on Vertigo, 345

Cadbury, Mr., Munificent Gift of, 390
Cagney, Dr., on Treatment of Disease by Electricity, 316
Caigen, Dr. F., on Diphtheritic Paralysis, 329
Calculi and Olive Oil, Biliary, 233
——— Renal, 188
Cameron, Dr. Spottiswoode, on Necessity of more Infectious Hospitals, 262
Campbell, Dr. C. M., on the Skin Diseases of Infancy, 125
Cancer, 59, 266
——— of the Tongue, 235
——— Some Probable Causes of, 94
——— Uterine, 157
——— Wards at the Middlesex Hospital, The, 100
Carbuncle and Boils, Treatment of, 265
Cardiff Infirmary, The Extension Scheme at, 121
Carr, Dr. W. L., on Salol, 61
Carter, Dr. Brudenell, and the Cure of the Insane, 89
Catarrh of the Stomach, Acute, 396
Cerealine Flakes, 380
Charitable Appeals and Charities, 97
Charities, Village, 289, 305
Charity Organization Society, 391, 405
——————————— on Hospital Accounts, The, 405
——————————— The "Black List" of the, 231
——— Fêtes, in Paris, 88
——— Reform Voting System, 40
Charity's Financial Outlook, 159
Charnwood Forest, New Convalescent Home in, 120
Chart, Danielson's, 365
——— Nursing, 10
Charteris, Professor, on the Impurities of Salicylic Acid, 395
Chest, Diseases of the, 345
Chester Hospital Saturday Committee, 278
——— Infirmary, 375
Chichester, Sanitation at, 106, 115, 152, 154
Children's Aid Association, Invalid, 99
——————— Dinner Society, The Destitute, 184
——————— Underclothing, 138
Chinaman, John, M.D., 83
Chloroform, The Hyderabad Commission on the Action of, 140, 185, 282
——————— Commission, Confirmation of the Conclusions of the Hyderabad, 120
Cholera, Prevention of, 203
Chorea, Pathology of, 314
Chorley, New Dispensary Building for, 214
"Christia" and Modern-Day Poultices, 28
Christmas Entertainments, 279, 310
Church Parades, 122
Clapham Maternity Hospital, 80
Clark, Sir Andrew, on The Constitution of Man, 35
Clergyman's Sore Throat, 204
Clifford, Edward, on Father Damien, 300
Cobbe, Miss, on Doctors as Centres of Infection, 129
Cocaine, Results of Using, 57, 104, 331
Coglitore, Dr., on Averting Cinchonism, 92
Collier, Dr. W., on Sensation in Animals, 24
Colman, Dr. Walter S., on Hyoscine, 93
Colon Flushing in Typhoid Fever, 107
Colotomy, 204
Colour Blindness, 362
Colouring Milk for Sale, 379
Committee, An Impeached, 367
Conscience, 49
Consumption, the Mount Dore Cure for, 72
Convalescent Home, Gift of Site for a, 390
——————————— The Gift of £100,000 to Found a, 223, 230
——————— Homes, Reports of, 40, 105, 342, 390, 391
Cooks and Cooking, 78
Co-operation in the Medical Profession, Need of, 138
Copeman, Dr. Monckton, on Detection of Human Blood, 28
Cornet, Dr., on Tubercle Infection, 155
Cornwall as a Winter Resort, 331
Cottage Hospital Wards, The Walls of, 207
——— Industries in Scotland, 48
——— Nurses and their Training, 268
Cough, Winter, 203
Coventry, A New Workhouse Infirmary at, 105
——————— New Workhouse Infirmary at, 359
——————— Hospital, Hospital Saturday at the, 104

Cremation and Urn Burial, 134
Creolin Formulary, The, 379
Crookshank, Prof., on Vaccination, 287
——— Mr., on Vaccination, 409
Croup, 300, 345
Cumberland Infirmary, Refusal of Committee to Increase Medical Staff of, 397
Curgenven, Mr., On Oil of Eucalyptus in Scarlatina and Other Infectious Diseases, 407
Cutis Laxa, 362
Cycling in its Relation to Health, 205
Cyprus Society, The, 273
Cystinuria, 362
Cystoscope, the Electric, 348

Daly, Dr. J. Bowles, on Chinese Doctors, 83
——— Travelling for Health, 118
Damien, Father, 300
——— Fund, The Father, 185
Dana, Dr. Charles L., on Injuries from Electricity, 172
Darlington Hospital, Donation of the Misses Pease to the, 72
Darwinism on the Stage, 150, 181
Daudet, M. Alphonse, Criticism of a play by, 150, 166, 181
Deaf, A Visit to a Training School for the, 306
Death Certificates and Unqualified Assistants, 6
——— Changes After, 44
Déjerine, M., on Muscular Atrophy in "Tabes Dorsalis," 155
Dengue in Europe, 217, 361, 407
Dentition, 377
Deptford Medical Mission, 185
Derby Hospital Managers and Doctors' Degrees, 280
——— Infirmary Rules and Miss Nightingale, 120
Derbyshire General Infirmary, The Eightieth Anniversary of the, 104
Dercum, Dr. F. X., on Spinal Injuries, 124
——— on Shock, 43
Devonshire Hospital, Hospital Patients at the, 246
Dewsbury and the Honorary Medical Staff, 120
——— Hospital, Testimonial to the Medical Staff of, 319
——— Infirmary, Service in aid of the, 246
Diabetes in Childhood, 234
Diagnosis, Difficult, 393
Dickinson, Dr. Howship, on Presystolic Murmur, 187
Diet, The Monotony of, 284
Dieulafoy, Dr. on the Treatment of Asthma, 408
Diphtheria, Death from, 247
——— House at Zurich, 338
——— Treatment of, 173
Diplomas, Doubtful, 375
Dipsomania and Hypnotism, 10, 22, 96
Disease, Refutation of charge that Hospitals perpetuate, 263
Disinfection and Life Seeds, Mr. Wynter Blyth on, 271
Disinfectants, A Novelty in, 251
Dislocated Shoulder, Operative Treatment of, 315
Dispensaries, Hospital Out-Patient Reform and Provident Dispensaries, 267
Dispensary Doctors at Tamworth, Treatment of the, 89
Dissection, The Paddington Board of Guardians on, 137
Distribution of Disease, The, 123
District Nursing Society, The Birmingham, 101
Doctor in History, The, 411
Doctor's Domestic Intellect, The, 399
——— Necessity for the Protection of, 360, 391
——— Pulpit, The—
A Fresh Start, 21
A Plea For the Disappointed, 291
A Sermon for the Kitchen, 357
A Suburban Medical Lecture, 179
A Sunday Lecturer, 387
Below Par, 3
Christmas, "Outward and Visible," 195
Colour Blindness, 275
Compromise, 37
"Confusion worse Confounded," 213
Development, 53
Elixirs and Inoculations, 323
Food and Force, 337
For Self; For Man, 165
Influenza Personally Conducted, 307
In Self Defence, 257
Life and Living, 85
Medical and Common Morality, 241
Nerves and Epidemics, 227
Out of Physic: Into What? 401
Professional Pressure, 67
"Then" and "Now," 131
The Moral Fruits of Darwinism, 149
Unstable Mental Equilibrium, 371
"Wonder" or "Form," 117
Doctors under Surveillance, Hospital, 33
Doll Show proposed at the New Hospital for Women, 344
Dollinger, Dr., on the Evacuation of Vertebral and Psoas Abscesses, 363
Donegal Industrial Fund, Sale of the, 200
——— Peasant-industries and Mrs. Ernest Hart, 312
Donnet, Dr. J. L., on the Causes of Leprosy, 11
Donnybrook Royal Hospital for Incurables, 215
Dore Cure for Consumption, The Mount, 72
Dover Hospital, Opening of Jubilee Wing of, 152
Doyle, Mr. Gaynes, on Ainhum, 27
Doyon, Dr. Maurice, on Accumulation in the Organism of Bromide of Potassium, 157
Dreadnought Seamen's Hospital, Evidence of World-wide Fame of the, 200
Dressings, Lister's New Antiseptic, 142, 188
Droitwich Workhouse, Improvements at, 89
Drunkenness, Habitual, 351
Dublin Hospital Sunday Fund, 404
——— Hospitals, The Out-Patient Departments of, 69
Dufferin Fund, The, 185
Dufferin's Fund, Lady, 306
Dujardin-Beaumetz, Prof., on Locomotor Ataxy, 76
Dumb to speak, Teaching the, 343
Dundee, Opening of the King's Cross Hospital at, 184
Durnford, The Right Reverend Bishop, on the Cesspools of Chichester, 115, 154
Dust and Disease, 364
Dying in 1795, The Method of, 386
Dysentery, Treatment of, 141, 346

Earlswood as It Is, 309
Ears, Noises in the, 407
Eastbourne, Proposed Maternity Hospital at, 24
East End Mothers' Home, 230
——— The First Festival Dinner of the, 295
Edinburgh Royal Infirmary, Annual Meeting of the, 247
Electric Light Installed at New York Hospital, 263
Electricity, 379
——— as a Medicine, Dangers of, 202
——— Injuries from, 172
——— Treatment of Disease by, 316
Electromagnet in Ophthalmic Surgery, The, 80
Ellison's Opportunity, Ralph, 301
Emigrants' Hospital at Ward Island, The, 175
Enlargements of Hospitals, 10, 40, 72, 105, 152, 200, 231, 375, 405
Enteric Fever, Acetanilide in, 394
——— Fever in India, 171
Entertainments for the Poor, Canon Farrar on, 231
Epidemiological Society, London, 189
Epidemics—Past and Present, 303
Epilepsy, Successful Operation for the Cure of, 246
——— Trephining for, 108
Erysipelas, Arrest of, 364
Ether Death Under, 9
Ethical Query, 45
Eucalyptus in Scarlatina and other Infectious Diseases, Oil of, 407
——— Some Forms of Malaria, The Oil of, 282
Exalgine—A New Pain Killer, 376
Examination, Lord Coleridge and Professor Henry Morley on, 170
Excipient for External Application, 409
Exeter Eye Infirmary and the Claims of the Working Men Contributors, 239
"Exmouth," Influenza on the Training Ship, 247
Expenditure, Startling Differences in Hospital, 183

Fallen, Female Mission to the, 104
Farringdon, Dr. J. M., on Alcohol in Medicine, 107
February, 274
Fees in Germany, Doctors', 380
Felkin, Dr., on Fœtal Malaria, 265
——— Mr. R. W., on Sensation in Europeans and Negroes, 41
——— Dr. R. W., on the Distribution of Disease, 123
Femur, Injuries of the Neck of the, 378
Fernie, Dr. W. T., on Herbal Simples, 197, 212, 226, 292, 321, 354
Fever Hospital, London, 169
Filaria, 13
Financial Outlook, Charity's, 159
Fish as Food, 205
Flat Foot by Massage, Cure of, 362
Fleming, Mr. John, Munificent Bequests of, 358
Fliessinger, Dr., on Renal Lesions, 156
Flower, Mr., and Algerian Hints for Tourists, 16
Flowers and Trees, Nutrition and Propagation of, 84
Flux at Illinois and Warsaw, Bloody, 73
Fœtal Malaria, 265
Fogs of London, 122
——— and Luxuries, Some Unpleasant Truths About, 133, 184
Food for Invalids and Infants, 266
Foot-Warmers in Parisian Cabs, 201
Foreign Bodies in the Eye, 44, 80
——— Doctors in France, 136
——— Medical Literature, 74
Foulis, Dr. James, on Obstructed Breathing from Anæsthesia, 283
Fox, Dr. F., on Strathpeffer Spa, 333
Foy, Dr. Geo., on Gurjon Oil in Leprosy, 11
Fractures, Ununited, 92
Franks, Dr. Kendal, on Antiseptics, 105
Frantzel, Dr. Otto, on the Temperature in Influenza,
Fraser, Dr. Thomas, on Methyl-Acetanilide, 395
Friends and Medical Missionary Work, The Society of, 105

Gairdner, Dr., on Aortic Aneurism, 11
——— Dr. W. T., on Drunkenness, 351
Garrod, Dr. A. E., on the Pathology of Chorea, 314
Gerhardt, Dr., on Rheumatism, 361
German Hospital Board and the Active Medica Staff, 334, 350
Giacict, Dr., on Silk as a Dressing, 298
Glanders, Death of Dr. Hoffman from, 73
Glasgow, Opening of the Victoria Infirmary at, 327
Godlee, Mr. Rickman J., on Hepatic Abscess, 330
Goitre, 345, 393
——— by Electricity, Treatment of, 408
——— Iodine, Treatment of, 235
Good, Dr., on Meat Diet, 299
——— Rev. P. H., on the Borderland of Madness, 22
Goodchild, John A., Chats at St. Ampelio, by, 301
Gordon, Surgeon-General, C. A., on Military Hygiene, 125
Gout, Latent, 313
Great Northern Central Hospital, Exhibition of Pictures in Aid of the, 214, 263
——— Report of the, 358
——— Subscription Dances in Aid of the, 136
Great Ormond Street Hospital for Children, The, 5, 57
Greene, Dr. Richard, on a Design for an Asylum Infirmary, 111
Grimsby Hospital, Reforms at the, 374
Guinness, Noble Gift of Sir Edward, 136, 138
Gurjon Oil and Leprosy, 11
Guy's, Improvements at, 41
——— Mr. Gladstone Opens the New Medical School at, 405

Hadden, Dr. W. B., on "A Subjective Sensation in the Mouth of Women," 345
Hägler, Dr. C., on Tetanus as a Communicable Disease, 233
Haig, Dr., on Albuminuria, 281
Hair Dye, 58
Half Madness and Responsibility, 26
Halifax Borough Hospital, 404
——— Infirmary, Recommendations of the Building Committee of the, 105
——— Workhouse, 391
Hamilton, Deputy-Surgeon-General J. B., on Enteric Fever in India, 171
Hammer Nose, Dr. Squire on, 75
Hampstead Home Hospital, 359
——— Provident Dispensary, 398
Hare, Dr. H. A., on the Effect of the Entrance of Air into the Circulation, 205
——— Injury to the Phrenic Nerve, 299
Harris, Dr. Thomas, on Phthisis, 313
Harrison, Mr. Reginald, on Chronic Suppurative Pyelitis, 204
Hart, Mr. Ernest, on Smoke Abatement, 57
——— Mrs. Ernest, and Donegal Peasants, 312
Haslam, Mr. W., on Surgical Cases, 172, 188
Hastings University School and the Great Ormond Street Hospital for Children, 57
Haviland, Mr. Alfred, on Cancer, 59
Hay Fever, 203
Health, The Christian Church and the Bodily, 38
——— Travelling for, 118
Healthy House, The A B C of a, 137
Helbing, Mr., on the Creolin Formulary, 379
Henoch, Professor, on Tracheotomy in Diphtheria, 298
Hepatic Abscess, 249, 394
——— The Surgical Aspect of, 330
Herbal Simples, The History and Capabilities of, 197, 212, 226, 292, 321, 354
Heredity, 10, 59, 80
Hernia, 11, 378
——— Cerebri, 108
Herpes Zoster, 377
Herschell, Dr. George, on Electricity, 379
——— Meat Diet, 173
Hicks, Miss Philippa, on the Hospital for Sick Children, 57
——— Mr. Rivers, and Penny Quinine, 379
Hœmorrhoids, 346
Hoffman, Death of Dr., 73
Homerton Asylums Board Hospital Ball, 294
Honorary Medical Staff, Proposed Recognition of the Services of an, 120

Hood, Dr., on Hypnotism, 281
Horne, Mr. Fletcher, on Fracture of the Patella, 378
Hornsey Local Board, New Sanitary Depôt of the, 184
Horse-Accident Prevention Society, The, 186
Horsebeef at Antwerp, 343
Horses, The Home of Rest for, 86
Hospital Abuse, 150, 243
——— Doctors under Surveillance, 33
——— Expenditure, Reflections on, 383
——— for Sick Children, Public Schools Maintain Cots at the, 137
——— Problem, The: I. Hospital Income and Expenditure, 353; II. What is Wanted, 370; III. Hospital Audit and Book-keeping, 385
——— Reports, 25, 72, 88, 89, 104, 120, 121, 136, 137, 152, 153, 168, 169, 184, 214, 215, 230, 231, 246, 247, 262, 278, 294, 295, 310, 326, 327, 342, 343, 358, 359, 374, 375, 390, 391, 404, 405
——— Saturday Fund, 279, 327
——————— Awards, The, 270
——————— Committee at Chester, 278
——————— Enrolled under the Companies Acts, 375
———————Committee of the Coventry and Warwickshire Hospital, The, 104
——— Sunday Fund, Amount of the, 9
——————— and Institutions for the Incurable, The, 254
——————— Annual Meeting of the, 184
——————— Report of the Metropolitan, 169
Hospital Workshop, The—
X In the Wards at Great Ormond Street, Hospital for Children, 5
XI In the Wards at the Royal Free, 45
XII The General Hospital at Birmingham, 54
XIII The Jaffray Suburban Hospital, Birmingham, 79
XIV The Queen's Hospital, Birmingham, 94
XV The Birmingham District Nursing Society, 101
XVI The Birmingham General Dispensary, 126
XVII The Poplar Hospital for Accidents, 158
XVIII King's College Hospital, 190
XIX, XX St. Luke's Hospital for the Insane, 206, 252
XXI The Richmond Union Infirmary, 316
XXII The Taunton and Somerset Hospital, 356
XXIII West of England Sanatorium, 397
Hospitals Association, The, 24, 168, 326
——————— and Miss Broadwood's Paper, The, 268
——————— Discussion at the First Meeting of the Seventh Session of The, 56
——————— Dr. Bristowe on the Work of The, 51
———————in Improving the Management of Hospitals, Influence of The, 210
——————— Mr. Burdett's Lecture before The, 353, 359, 370, 385
——————— Opening of the Seventh Session of The, 24, 51
——————— New Members of The, 374
——————— Remarks on Mr. Wynter Blyth's Lecture before The, 271
——————— Persons Present at Mr. Wynter Blyth's Lecture on Disinfection before The, 200
——————— Persons Present at the Fourth General Meeting of The, 263
——————— Persons Present at the Reading of Sir Edmund Currie's Paper before The, 390
——————— Sir William Moore's Lecture on Leprosy and Leper Houses before The, 143
——————— The Ambulance Branch of The, 168
——— Attacked in the *Liverpool Post*, 295
——— The Christian Origin of, 290
——— The Support of, 49
Howell, Constance, "A More Excellent Way" by, 300
Housing of the Poor, Sir E. Guinness's Gift for the Better, 136, 138
Huchard, M., on Morphia Injections, 93
Human Blood, Detection of, 28
Humour, The Speaker on, 186
Hunter, Dr. William, on Pernicious Anæmia, 107, 313
——————— Transfusion of Blood, 28
Hyderabad Chloroform Commission, The, 140, 185, 282
——— New Hospital for, 327
Hydrophobia, 329
——— Old Cures for, 198
Hygiene, Military, 125
——— Supplement au Journal d', 242
Hyoscine, 157, 171, 281
Hyoscine as a Sedative, 93
———and Mr. Tooth, 8, 10, 22
———at Helsingborg, 231
———Medically Considered, 335
———or Mesmerism, 69
———*v.* Dipsomania, 96
Hysteria in Dogs, 41
———in the Male, 187
Hysterical Amaurosis, 249

Ice, Cases treated by, 76, 109, 346
Illingworth, Dr., on Antiseptics *v.* Plain Water, 232
——— C. R., on Scarlet Fever, 173
Immisch Thermometer, The, 93
Incompatibles, 250
Incurable, The Hospital Sunday Fund and Institutions for the, 254
Incurables, The Manchester Hospital for, 136
Inebriate Women and Girls, 392
Inebriates, St. Raphael's Home for Women, 136
Infantile Mortality in London, 57
Infants' Diet, 347
Infection, Dr. Willoughby on, 406
Infectious Diseases Act and St. George's, Hanover Square, 9
——— Increased Hospital Accommodation Necessary Owing to, 262
———Removal of Persons Suffering from, 168
———Doctors, Miss Cobbe on the Notification of, 129
———Hospitals, Noticeable Increase in, 200
Infirmaries, Provision for Sick Nurses in, 390
Influenza, Death from, 310
——— The Epidemic of, 170, 200, 202, 207, 215, 217, 230, 232, 242, 247, 278, 296, 328
——— The Temperature in, 297
Inhalatory Treatment of Phthisis, 59
Insane, The Hospital for the, 324, 388
——— Story of the, 4
Insanity in England, Statistics of, 381
——— The Causes of, 10
——— Cure of, 89, 405
Insect Powders, 38
Insects in Drugs, 346
Insomnia, 395
——— No More, 156
Insurance, The German System of National, 15
Intellect, The Doctor's Domestic, 399
International Hospital at Naples, 215
Introductory Addresses at the Medical Schools, 29
Invalid Children's Aid Association, 99
———Transport Corps, 57
Invalids' Dinner Table at University College Hospital, 405
Iodides for Scrofulous Children, 156

Jackson, Mr. Vincent, on Lithotomy, 60
Jaffray Suburban Hospital, Birmingham, The, 79
January, 211
Jeaffreson, Mr. C. S., on the Electro-magnet in Ophthalmic Surgery, 80
——— Nursing Chart, 10
Jefferson, College, Philadelphia, Successful operation at, 246
Jervis Street Hospital, Annual Meeting of the, 243
Jeye's Automatic Distributor, 251
Jones, Dr. Mary, on Ovarian Disease, 59
——— Dr. Robert, on Trephining for Epilepsy, 108
Joule, Dr., Death of, 41
Jubilee Institute for Nurses at Edinburgh, First Report of the, 121
Judicial Decision important to Charities, 296

Keith, Leslie, "Ralph Ellison's Opportunity," by, 301
Kerslake, Mr. F., on Compulsory Muzzling of Dogs, 73
Khakanof, Dr., on Antifebrin, 92
Kiesselbach, Dr., on Noises in the Ears, 407
Kilhampton Home, Inquiry at, 215
King's College Hospital, 190
——— Cross Hospital, Dundee, Opening of the, 184
Kingzett, Mr. C. T., on Antiseptics, 44
Kinnear, Dr., on Cases treated by Ice, 76
Kirmes at Buffalo, A., 302
Knee-joint Disease, 250
Knott, Dr. J. F., on Purpura, 43
———on Bleeding, 75
Kohlmann, Dr., on Communicability of Cerebrospinal Meningitis, 155
König, Dr., on Colour Blindness, 362
Kossobudski, M., on Hæmorrhoids, 346
Kraske-Riedel method of arresting Erysipelas, 364
Küster, Prof., on Paracentesis in Empyema, 363
——— the Treatment of Abscesses in Cavities with rigid walls, 363

Lactotherme, The, 348
Ladies' Charity School for Training Young Girls as Domestic Servants, 311
——— Suggested Medical Missionary Training Institution for, 104
Laing, Peter, 231
Lancaster Infirmary, 24
Lanceraux, Dr., on Phthisis, 262
Landerer, Dr., on Balsam of Peru in Phthisis, 407
——————— the Cure of Flat Foot by Massage, 362
Larkin, Mr. Charles, on Removal of Accessory Limb, 60
Lead Poisoning in Sheffield, 264
Lectures, People's, 404
Leeds Workpeople's Hospital Fund, 311
Lees, Dr. D. B., on the Ice Treatment, 109
Leper Hospital at Bombay, Gift to Found a, 342
Lepers at Robbin Island, 9
——— in Calcutta, 312
——— of Molokai, Departure of Miss Fowler to the, 263
Leprosy and Leper Houses, 143
Leprosy at New Caledonia, Outbreak of, 326
——— Causes of, 11
——— Fund, The, 185
——— Gurjon Oil and, 11
——— Rev. W. T. McCormick on the Noncontagious Nature of, 294
——— The Bombay Medical and Physical Society on, 139
——— Cure of, 297
——— Etiology of, 187
Lewisham Workhouse, 294
Lewtas, Dr. J., on Traumatic Subclavian Aneurism, 12
Ligature, The Temporary, 330
Limbs, Artificial, 364
Lissauer, Dr., on Mental Blindness, 297
Lister, Sir Joseph, on the Operative Treatment of Dislocated Shoulder, 315
Lister's New Antiseptic Dressing, 142
Lithotomy, 60
Liverpool Dispensaries, Annual Report of the, 359
———Street Accidents, 137
Loch, Mr. C. S., on Charitable Appeals, 97
Lockjaw resulting from Vaccination, 72
Lockwood, Mr. C. B., on Hernia, 12
London Empty, 1
——— Pauperism in, 225, 244
——— The Bishop of, on the Moral Basis of Private Property, 322
Loreta's, Prof., Treatment of Ununited Fractures, 92
Lotteries as Aids to Hospital Funds, A Suggestion Concerning, 280
——— Charity, 238
Lucas, Mr. Clement, on Necrosis of the Jaw, 60
Lunacy Amendment Act, 121
——— First Annual Report of the State Commission in, 342
——— Service, The Irish, 264
Lunatic Asylums in Scotland, The Report of Commissioners on, 10
Lunatics, Pauper, in Licensed Houses, 296
Lung, Antiseptic Drainage of the, 91

Macfarlane, Dr. A. W., on Insomnia, 395
Mackenzie, Dr. Conll, on Changes After Death, 44
Madden, Dr. More, on Uterine Cancer, 157
Madness, The Borderland of, 22, 26
Magnan, Dr., on Absinthe, 155
Maidstone, Munificent Gift to the West Kent Hospital at, 391
Malagola and Montalti, Drs., on Antiseptic Drainage of the Lung, 91
Malaria, Fœtal, 265
——— *v.* More Recognizable Causes of Disease, 189
Malarial Fever, Treatment of, 233
Male, Hysteria in the, 187
Malingering, 281
Man, The Constitution of, 35
Manchester Hospital Sunday and Saturday Funds, 153
Manley, Dr. T. H., on Temporary Ligature, 330
Manton, Dr. J. A., on Hypnotism or Mesmerism, 69
Manufacturing Pursuits, Causes of Diseases in, 40
Manwell, M. B., "Dad's Dorothy," by, 333
Marano, Dr., on the Treatment of Goitre by Electricity, 408
March, 340
Market Drayton, Proposed Cottage Hospital at, 405
Marston, Surgeon-General, on Hepatic Abscess, 394
Martin, Dr. E., on Injury to the Phrenic Nerve, 299
——— Sidney, on Proteid Poisons of Jequirity, 44
——— Dr. Stephen C., on Vaccination, 378
Matrons, The Powers of, 58
McCormick, Rev. W. T., on Leprosy, 294
McIlvaine, Mr. A. Robinson, on Adulteration of Drugs, 315
Meat Diet, 173, 299
Medical Council on Registration of Midwives and Nurses, The General, 163
——— Literature, Foreign, 7
——— Missionary Society, Edinburgh, 104

Medical Missionary Work by the Society of Friends, 105
——— Practitioner, The Oppression of the, 166
——— Science, The Popularization of, 113
——— Staff and Hospital Boards, The Active, 334, 350, 381
Meldon, Dr. Austin, on the Pasteur System, 104
Men, Openings for Young, 74
Mendicity Society, Memorandum of the, 185
Meningitis, Communicability of Cerebrospinal, 155
Mental Blindness, 297
Mercury, Novel Use of, 346
Mesmerism, Hypnotism, or, 69
Metropolitan Asylums Board, Reports of the, 247
——— Report of the Ambulance Committee to the, 168
——— Hospital, The, 404
——— Annual Meeting of the, 295
——— Provident Medical Association, The, 88
Microbes, 27, 75, 284
——— and Poisons, 13
Middlesex Hospital, The Cancer Wards at the, 100
Mikuliez's Operation, 379
Mild Weather, Danger of, 122
Milk for Infants, 332
—— Poisoning, 298
Milner, Mr. James, on Hysterical Amaurosis, 249
Mind, The Medication of the, 65
Montreal, Death Under Ether at, 9
Montrose Asylum, Unsuccessful Plan for the, 111
Moon, Dr., The Case of, 90
Moore, Dr. W., on the Oppression of the Medical Practitioner, 166
——— Sir W., on Malaria, 189
——— William, on Leprosy and Fever Houses, 143
Morgan, Mr. A. Kinsey, on Bournemouth as a Health Resort, 333
Morphia Injections, 93
Murmur, Presystolic, 187
Murray, Dr. W., on Renal Calculi, 188
Murrell, Dr. Wm., on Winter Cough, 203
Musser, Dr. J. H., on the Treatment of Ague, 282
Muzzling of Dogs, A Deputation on the, 184
——— Mr. Kerslake on, 73

Naphthol in Dyspepsia, 394
National Insurance, the German System of, 15
Navy, the Health of the, 311
Necrosis of the Jaw, 60
Netley, Nursing at, 58
Newcastle College of Medicine, Opening of, 25
New Convalescent Home for Leicestershire, 120
—— Hospitals, 40, 73, 105, 121, 184, 185, 200, 310, 326, 327, 358, 359, 375, 405
——— in 1889, 200
—— Journalism and the Hospitals, The, 391
New York Hospital, Description of a, 41
——— News from, 246
Night Medical Service, A, 230
Nightingale, Miss, on the Derby Infirmary Rules, 120
Nigrisoli, Dr., on Professor Loreta's Treatment of Ununited Fractures, 92
Nile, Winter Climate of the, 347
Norbury, Deputy Inspector General, on Alum Injection for Dysentery, 346
Norfolk and Norwich Hospital Sunday Fund, 214
——— Hospital, History of the, 310
North-West London Hospital, Lottery at the, 152
Notification of Infectious Diseases Act and Birmingham, 17
Nunn, Mr., on Malingering, 281
Nurse-Training School Authorities and Registration, 147, 180, 229
Nurses, National Pension Fund for, 47, 255

Obesity, 11
Obstetrical Society for the Northern Counties, 214
Omaha, Red-tapeism at, 294
Operations in Hospitals, The Right to Perform, 372
Operations, Minor, 172
Ophthalmia Neonatorum, 233
Orphan School, Annual Meeting of the Royal Seamen's and Marines', 136
Otto, Dr., on Pleurisy, 297
Out-Patient Reform and Provident Dispensaries, Hospital, 267
Out-Patients at Dublin Hospitals, 89
Ovarian Disease, 59
Ovariotomy and Listerism, 265
Paracentesis in Empyema, 363
Paraffinum Molle, 365
Paralysis, Alcoholic, 188
——— Diphtheritic, 329
Parish Doctor, The, 170
Parke, Dr., and Stanley, 137
Parker, Dr. W. Thorton, on the Cause of Typhoid Fever, 265
——— Mr. Rushton, on Trephining for Cerebral Tumour, 249
Parsons, Dr. George, on Sea Sickness, 60
——— J. Inglis, on Cancer, 266

Pasteur, M., 390
——— and the Australian Rabbit Commission, 406
——— System, Dr. Austin Meldon on the, 104
Pasteurism, Sir Henry Roscoe on, 42
Pastimes as Aids to Hospital Funds, Popular, 116, 237, 258, 276
——— Proceeds from, 136
Patella, Fractured, 27, 378
Pauperism in London, 225, 244
Payne, Dr. J. Frank, on Acne, 377
Pearse, Dr. W. H., on Phthisis, 107
Pedley, Mr. R. D., on Puerperal Fevers, 329
Pelican Club, The, 248
Penitentiary Association, The Church, 402
Penny Quinine 379
Pension Fund for Nurses, A Donation to the, 374
——— Second General Meeting of the, 47
——— The Extension of the, 255
Penzoldt, Dr., on a True Stomachic, 408
Petit, Hon. Sir Dinshaw Manockjee, Munificent gift of, 342
Pfeiffor, Dr., on Green Stools in Infants, 234
Philadelphia German Hospital, 374
Philanthropist's Vade Mecum, The, 218, 236
Philanthropy, Growth of practical, 209
Philippo, Dr., on the cure of Leprosy, 297
Phrenic Nerve, Injury to the, 299
Phthisis, 59, 107, 262, 313, 407
Physicians, The Fire extinguishing apparatus at the Royal College of, 310
Pierce, Dr. W., on the Treatment of Enteric Fever by Acetanilide, 299
Pines at Bournemouth, Among the, 148
Playfair, Sir Lyon, "Subjects of Social Welfare," by, 301
Pleurisy, Compression of the Thorax for relieving the pain of, 297
Plumbism, 264
Pneumonia, Discussion at the Harveian Society on the Treatment of, 109
Poisons and Microbes, 13
Polk, Dr. W. M., on Medicine and Socialism, 315
Polypus, Mr. Haslam on Malignant, 188
Poor in Workhouses, Classification of the, 81
——— Mr. H. C. Burdett's Scheme for helping the London, 101
Popularisation of Medical Science, The, 113
Pouchet, M., on Accumulation of Arsenic in Spongy Bone, 234
Poultices, Modern-Day, 28
Posthumous Gifts to Charity, the Squandering of, 412
Powell, Dr. Douglas, on Aortic Aneurysm, 281
Practitioner's Outlook and Retrospect, The, 11, 27, 43, 59, 75, 91, 107, 123, 139, 155, 171, 187, 203, 233, 249, 265, 281, 297, 313, 329, 345, 361, 377, 393, 407
Presystolic Murmur, 187
Princess of Wales and Nurses' Pensions, The, 376
Private Property, The Moral Basis of, 322
Probationers at Cottage Hospitals, Paying, 104
Professional Pressure, 67, 121, 168, 175
Progressive Paralysis, 27
Providence Hospital, St. Helen's, 185
Provident Medical Association, Metropolitan, 88
Puerperal Fever at the Philadelphia Lying in Charity, 343
——— Fevers, 329
Purpura, 43
Puzey, Mr. Chaney, on Progressive Paralysis, 27
Pyelitis, Chronic Suppurative, 204

Quack Doctors, 19
Queen's Hospital, Birmingham, The, 40, 94
Quinine, 92
——— Penny, 379

Railway Officers and Servants' Association and Railway Orphan Fund, The, 169
Railways, Condition of London, 74
Registration of Midwives and Nurses, The General Medical Council on the, 161, 163, 177
——— Nurses, Memorial of Nurse-Training School Authorities against the Proposed, 147, 180, 229
Reid, Mr. Peter, Munificent Gift of, 223, 230
Reimer, Dr., on Scarlatina Treatment and Results, 91
Religious Orders from Italian Hospitals, Expulsion of, 120
Renal Calculi, 188
——— Lesions, 156
Rentoul, Dr., on Hospital Out-Patient Reform and Provident Dispensaries, 267
Rentoul's Scheme, The General Practitioners on Dr., 127
Reports, Convalescent Homes, 40, 105, 342
——— Hospital, 25, 72, 88, 89, 104, 120, 121, 136, 137, 152, 153, 168, 169, 184, 214, 215, 230, 231, 246, 247, 262, 278, 294, 295, 310, 326, 327, 342, 343, 358, 359, 374, 375, 390, 391, 404, 405
Respirator, The Derbyshire, 189

Reviews:—
Algerian Hints for Tourists, 16
A More Excellent Way, 300
A Text-book of General Therapeutics, 142
Chats at St. Ampelio, 301
Cremation and Urn-Burial, 134
Dad's Dorothy, 333
Father Damien, 300
History and Pathology of Vaccination, 409
Insomnia, 395
James Vraille, 381
Ralph Ellison's Opportunity, 301
Subjects of Social Welfare, 301
The A B C of a Healthy House, 137
The Doubts of Dives, 196
The Review of Reviews, 396
The Year Book of Treatment for 1890, 365
Two Health Resorts, 333
Rheumatism, Diagnosis and Treatment of the Several Forms of, 361
Rhinitis, 249
Rhinophyma, Dr. Squire on, 75
Richmond Union Infirmary, 316
Ringer, Dr. Sidney, on Hydrophobia, 329
Ripley Hospital, Lancaster, New Chapel at, 137
Rivington, Mr. Walter, on Stricture, 43
Robben Island, The Lepers of, 9
Roberts, Sir Wm., on Latent Gout, 313
Robertson, Sir William Tindal, 25
——— Stewart Hospital, Rothesay, Annual Meeting of the, 214
Robinson, W., on Cremation and Urn-Burial, 134
Robson, Mr. Mayo, on Transfusion, 43
Rochford Hospital for Infectious Diseases, Mismanagement at, 73
Roscoe, Sir Henry, on Pasteurism, 42
Rose, Dr. A., on the Diagnosis of Sickness in the Street, 281
Rosenburg, Dr. Siegfried, on Biliary Calculi and Olive Oil, 233
Ross, Dr. James, on Alcoholic Paralysis, 188
Rotunda Hospital, Election to the Mastership at the, 105
Royal Free Hospital, Annual Meeting of the, 326
——— Ball, 168
——— Description of the, 45
——— Vindicated from a False Charge, 84
——— Hospital for Diseases of the Chest, 31, 33, 56, 61, 145, 152, 176
——— Seamen's and Marines' Orphan School, Meeting of the, 136
Royalty at Hospitals, 390
Rumbold, Dr. T. R., on Rhinitis, 249

Saccharin, 346
Salicylic Acid, Impurities of, 395
Salisbury Provident Dispensary, 279
Sandgate Convalescent Home, Enlargement of, 10
Sanitation at Chichester, 106, 115, 152, 154
——— in America, 8
Sanitary Aids to House-Hunters, 391
——— Depôt at Hornsey, New, 184
——— Institute, The Report of the, 263
Saturday Fund, Enrolment under the Companies Acts of the Hospital, 375
——— Meeting of the Hospital, 327
——— Hospital Fund Awards, The, 270
Saundby, Dr. Robert, on Acute Catarrh of the Stomach, 396
Savill, Dr., on the Winter Climate of the Nile, 347
Scandinavia, Notes from, 198
Scarlatina Treatment and Results, 91
Scarlet Fever Aborted by Biniodide of Mercury, 173
——— Increase of, 25
——— Influenza, Dengue, and, 217
——— Skin Disinfection in, 14
Schaffle, Dr. A., on the Quintessence of Socialism, 70
School of Cookery, National Training, 78
Schools and the Maintenance of Cots at the Hospital for Sick Children, 137
Scientific Knowledge, Diffusion of, 42
Scientists and their Rewards, 8
Scleroderma, 364
Scrofula, Mr. F. Treves on, 76
Scrofulous Children, Iodides for, 156
Scurvy, 27
Sea-Bathing Infirmary, Margate, Centenary of the Royal, 169
Seamen's Hospital, Greenwich, Annual Court of the, 327
——— Cookery Lectures at the, 200
——— Evidence of World-wide Fame of the, 200
Sea-sickness, 60
Secretaries of Hospitals and their Limitations, 145
Seifert, Dr. Otto, on Cutis Laxa, 362
Sensation in Animals, 24
——— Europeans and Negroes, 41
Sequah—A Modern Mountebank, 392
Sewerage Works at Sydney, New, 120
Shadwell Maternity Home, 89
Sheffield, Hospital Sunday at, 310

Sickness in the Street, Diagnosis of, 281
Silk as a Dressing, 298
Simples, A Few Old, 132
——— The History and Capabilities of Herbal, 197, 212, 226, 292, 321, 354
Skin Diseases of Infancy and Early Life, 125
Sleeplessness, Hereditary, 202
Small-pox, Discovery of the Bacillus of, 374
Smells, Bad, 154
Smoke Abatement, Mr. Ernest Hart on, 57
Smokeless, Process Rendering Coal, 26
Snell, Mr. Simeon, or Foreign Bodies in the Eye, 44
Social Welfare, Subjects of, 301
Socialism and Medicine, 315
———Modern, 70
Somnolency, Two Remarkable Cases of, 120
Southend Local Board, Proceedings at the, 153
Southsea, Home for Sick Children, 391
Special Hospitals. Small, 366
Specific Diseases, 406
Spender, Dr. K., on the Use of Antimony, 347
Spinal Injuries, 124
Spohr, Dr. Arthur E., on the Treatment of Diphtheria, 173
Stafford, New Hospital for, 73. 185
Starr, Dr. Louis, on Infants' Diet, 347
State Charities' Record, 359
———Subsidies to Dublin Hospitals, 214
Stern, Dr. R., on Dust and Disease, 364
Sternberg, Surgeon-Major G. M., on the Treatment of Yellow Fever, 109
Stift on Saccharin, 346
St. John Ambulance Association at Coventry, 326
———Centre at Paris, 169
St. John's Hospital for the Skin, 90
——— and the Hospital Saturday Fund, 286
——— The Accountants' Report on the, 279
St. Luke's Hospital for the Insane, 206, 252
St. Mary's Hospital, The Convalescent Fund at, 358
Stockwell, Dr. G. Archie, on Brain Wounds, 141
——— Hernia Cerebri, 108
Stomachic, a True, 408
St. Pancras Workhouse, Proposed Removal of, 328
St. Raphael's Home for Consumptive Poor at Worthing, 57
——— Women Inebriates, 136
——— A Dresser at, 312
——— Hospital, Election of Mr. J. Gadesden Wainwright as Treasurer of, 404
Strangulated Herniæ, Reduction of, by Coughing, 362
Strangulation, Intestinal, 314
Strathpeffer Spa, 333
Strike Relief Fund, Balance-sheet of the, 201
Strother, Mr. C. J., on Insects in Drugs, 346
Suffolk General Hospital, Peace at Last at the, 375
———Quarrel at the, 73, 136
Sulphonal, 124, 156, 331
——— Dangers of, 408
Sunderland Infirmary, Opening of New Wing at, 231
Surgery, Recent Progress in, 25
Surgical Cases, 172, 188
——— Dressings, 188
Sussex County Hospital, Alleged Mismanagement at the, 367
Syphilis, 314

Tadanoü, Surgeon-Major, on the Prevention of Cholera, 203
Talysin, Dr., and Scurvy, 27
Tamworth and the Dispensary Doctors, 89
Taunton and Somerset Hospital, The, 89, 356
Taylor, Dr. B. W., on Syphilis, 314
———James, on Hyoscine, 93
Tea, How to Make, 26
——— Revolutions in, 77
Temperature, 377
———A Case of High, 310
Terillon, Mr., on the Treatment of Goitre by Iodine, 235
Tetanus, a Communicable Disease, 233
Therapeutics, A Textbook of General, 142
Thermometer, The Immisch, 93
Third-class Carriage, The, 259
Thomas, Mr. Hugh, on Injuries of the Neck of the Femur, 378
——— Owen, on Fractured Patella, 27
Thompson, Dr. E. Symes, on Heredity, 80
Thomson, Mr. W., on Knee-joint Disease, 250
Tight Lacing, Monkeys and, 360
Tobacco, A New Danger from, 376
——— The use of, 141
Tonsil Slitting, 156
Tooth, Mr., as a Hypnotist, 8, 10
Torbay Hospital, Entertainment in Aid of the, 230
———The Matron of, on the Needs of the, 121
Town Dweller, The, 339, 369
———Noises, 9
Toys for London Hospitals, 185
Tracheotomy in Diphtheria, 298
Transfusion of Blood, 28, 43
Travelling for Health, 118
Trephining for Cerebral Tumours, 249
——— Epilepsy, 108
Treves, Mr. F., on Scrofula, 76
Tubercle Infection, 155
Tuberculosis, Iodoform in Articular, 363
Tuckey, Dr. C. L., on Cases Treated by Hypnotism, 171
Tuczek, Dr., on Antipyrin, 92
Tupper, Martin, 154
Turner, Sir William, on Heredity, 59
Typhoid Fevers, 107
——— Fever, Cause of, 265
Tyson, Dr., on Atropine, 109.

Underclothing for Children, 138
Underwood, Dr., The Opinions of, 248
University College Hospital, Invalids' Dinner Table at, 403
Unqualified Assistants and Death Certificates, 6

Vacation, More Voyages in, 284
Vaccination, 378
——— Antiseptic After-Treatment of, 408
——— History and Pathology of, 409
——— Is it a Fraud? 287
——— Lockjaw Resulting from, 72
Vade Mecum, The Philanthropist's, 218, 236
Vandenabule, Dr., on the Reduction of Strangulated Herniæ by Coughing, 362
Variation of Symptoms from the Type, 282
Varix, Operations for, 12
Ventilation for Hospitals and Sanatoriums, Antiseptic, 68
Ventnor Consumption Hospital and the Judges, 296
Verneuil, Professor, on the Treatment of Carbuncle and Boils, 265
Vertigo, 345
Victoria Hospital for Children, Appeal by the, 137
Village Charities:
1. Hard-and-Fast Rules of Distribution, 289
2. Occasional Gifts, 305
Vivisection, Mr. Browning and, 186
Volkmann, Professor, on Cancer of the Tongue, 235
Voltaic Current as a Cure for Cancer, 266
Vomiting, Prevention of, 364

Waifs and Strays Society, Quarterly Statement of the, 72
Wallis, Mr. Percy E., on Herpes Zoster, 377
Walls of Cottage Hospital Wards, the, 207
Ward Island, The Emigrants' Hospital at, 175
Wastefulness in Hospitals, Unwarrantable charge of, 296, 318, 333
Water-gas, deaths from, 201
Waterlow, Sir Sydney, and his gift to London, 120, 122
Way, A More Excellent, 300
Weaver, Mr. John J., on the Abuse of Hospitals, 243
Webb, Mr. J. H., on Minor Operations, 172
Webster, Mr. A. W., on Popular Pastimes as aids to Hospital Funds, 116, 237, 258, 276
Wessinger, Dr. John A., on Cocaine, 331
West of England Sanatorium, 397
White, Dr. W. Hale, on Therapeutics, 142
Whitehead, Sir James, Eulogy on, 106
Whitworth Trustees and Owen's College, 24
Wilding, Dr., on Antifebrin, 13
Williams, Dr. Watson, on Chronic Laryngitis, 204
Williamson, Dr., on Trephining for Epilepsy, 108
Willis, Mr. D. Armstrong, on Monotonous Diet, 284
——— W. Armstrong, on Cycling in its relation to Health, 205
Willoughby, Dr., on Specific Diseases, 406
Wilson, Dr., at the Sanitary Congress, 9
Winslow, Dr. Forbes, opens a new hospital, 391
Witham, Dr., on Microbes, 284
Within the Wards:
Some Probable causes of Cancer 94;
Uterine Cancer, 157
Wives of Eminent Men, 344
Wolfe, Dr. J. R., on Transplantation of Rabbits' Conjunctiva to the Human Eye, 314
Wolseley, Lord, on Women Nurses for the Army, 201
Woman—Is She the Lesser Man? 90
Women, a subjective sensation in the mouth of, 345
Wood-wool Company's Preparations, The Sanitary, 410
Woods, M.D., Hugh, and his unsubstantiated charges against Hospitals, 296, 318, 333
——— on Hospital Abuse, 150
Worcester, Activity at, 326
——— Dispensary, Difficulties at the, 40
Words of Consolation:
A Happy New Year, 210
Forgiveness of Sin, 18
Freedom, 336
Fruit, 146
Gifts to Christ, 240
Good Friday, 400
Goodwill Towards Men, 178
Joy, 50, 98
Knowing Christ, 224
Light, 130
Long-Suffering, 66
Man's Wants, 304
Mourning, 384
Paradise, 82
Peace, 2
Perfection, 320
Perseverance, 368
Prayer, 98
Repentance, 352
The Babe of Bethlehem, 194
The Dignity of Work, 272
The Infancy of Christ, 256
The Sure Foundation, 34
Thick Darkness, 162
Thoughts, 114
Unsatisfied, 288
Workhouses, Classification of the Poor in, 81
Working Boys of London, Homes for, 152
———Men and Hospital Committees, 382
——— their Contributions to Hospitals, 239
Workmen's Aid Society, the Metropolitan Destitute, 253
Workpeople's Hospital Fund, Bradford, 311

Yellow Fever, the Treatment of, 109
Yeo, Dr. Burney, on Food for Invalids, 266
——— Milk for Infants, 332

Zenana Medical College, Annual Meeting of the, 201
Ziemssen, Dr. V., on Hynoptism, 157
Zurich, The Diphtheria House at, 338

### THE SPECIAL NURSING SUPPLEMENT (MIRROR).

Aberdeen Royal Infirmary, Miss Lumsdens, Work at, xxv
Adelaide Letter, Mary, lxxiii
Addenbrooke's Hospital, xiii
Aged Nurses, Work for, liii
Alcott, Miss, Condemns Nurses for Living Unhealthy Lives, xxxvii
———Life and Letters of, xiii
America, Nursing in, lxxxiii
American Notes, xlii, lxxiii, lxxxiii, cv
———Nurses, Gift to, lxxxv
Answers to Examination Questions, ii, xxvi, xxx, xlvi, lv, lxxv, lxxix, xci, xcv, cviii
Army Nurses, xlv
Ashton Nursing Association, lxxiii
Asylum Attendants, ix, xxxi, xli, lv
Asylums Board on Matrons, The, xxv
——— Sisters' Dresses, The, xxxiii
Australia, Notes from, xiv, li., lxxxiii, cvi
———Nurses in, vii

Berwick, Nursing at, xciii
Bolton Workhouse, Scandal at, xli, liii
Bookshelf, The Nurses'—
District Nursing, lxxxvii
Manual of Family Medicine and Hygiene for India, A, l
Manual of Nursing, Medical and Surgical, A, lxvi
Bookshelf, The Nurses'—
Masso-Therapeutics, xliii
Minor Surgery and Bandaging, l
Nursing for Sixpence, lxxxvii
Nursing Questions and Answers, lxxxvii
Obstetric Nursing, lxxi, lxxiv
Recollections of a Nurse, The, xcv
Toilers in London, xliii
Bournemouth Nurses' Institutes, vii.
Boy, A Hospital, xxxiv
Brassey Holiday Home, lxxvii, cv
Bristol, New Nursing Home at, xxxiii
British Nurses' Association and Registration Scheme, Refusal of the Hospitals to Countenance the, lxix

British Nurses' Association, its Scheme of Voluntary Registration, lxxx. lxxxi
—— Appeals to Hospitals, The, xxxv
—— Conversazione, The, xlv
—— Journalistic Methods of the xlix
—— Weekly Commissioners on Toilers in London, The, xliii
Burton-on-Trent Infirmary, xxi
Butterworth Medals at Guy's, Presentation of the, lxiv

Canning Home for Nurses, vii
Cap, A New, xxix
Cardiff Docks, The Hospital Ship "Hamadryad" at, xci
Case Book, A Nurse's, lvii, lxv
—— Taking, ii, viii
Cases, Between, xcix
Certificates and Registration, li
Chicago, Touching Tribute to the Late Matron of the St. Luke's Free Hospital, civ
Chichester Infirmary, Improvements at the, xxix
Children, The Management of, xxvii, xxx, xxxv
Christmas Decorations, xvii
—— Entertainments, xlix, lv, lx, lxii, lxvii, lxxi, lxxiii
Church Army Nurses, The Training of, xlv, lvii
Continent, Nursing on the, xxxvii
Co-operation, Nurses', xxvii, xxxix, xliv, lxxxi
Cottage Hospital Matrons, xv, xviii, xxiii
—— Hospitals and the Pension Fund, lxix
—— Nurses, lxi
Coventry District Association, lxxxi
Craven, Mrs. Dacre, on District Nursing, lxxxvii

Decoration, Ward, xxxviii
Detractor of the Pension Fund, A, lxv
Dispensing, ci
District Nurses, Ladies as, xiii, xxiii, xxvii, xxxi, xxxix
—— Nursing, xxxi, xxxiii, xxxvii, xlv, liii, lxi, lxxxi, lxxxv, lxxxvii, lxxxix, xciii, xcvii, ci, cv
Doll Competition. The. iii, xiii, xxix, xxxix, xlii, xlvi, lii, lvii, lxix, lxxix, xciii
—— Show, A Journalist's Account of the, xli
Dresses in Fever Hospitals, Sisters', xxxiii
Drew, Mena, on Nursing, lxxxvii
Dundee, District Nursing at, xxxiii

East London Nursing Society, lxxxix
Edinburgh, Nursing at, lxv
Electricity, Medical, xcv
Emigration, ci
Examination Questions, Answers to, ii, xxvi, xxx, xlvi, lv, lxxv, lxxix, xci, xcv, cviii
Exeter Workhouse, Scandals at, i

Finches, Three, xxvi
First Experience. A, xliii, lxxxvii
—— Thousand, Honours for the, xcvii, cv

Glasgow Institutions, xxi
—— Training Home, xciii
Grumbling Letters, lvii, lxv

Hands, Care of the, liii
Hants Infirmary, New Nursing Scheme for South, lxv
Heanley, Miss, on Certificates and Registration, li
Heart and Paralysis Hospital, Soho Square, Entertainment at the, ci
Heath, Mr. Christopher, on Minor Surgery and Bandaging, l
Home Nurses at Grimsby Hospital, xiii
Hope Infirmary, Scandals at the, xvii, xxxi, xxxix
Hotel Dieu, The, xiii
Humphry, Dr. Laurence, on Nursing, Medical and Surgical, lxvi

Ideal Nurse, My, xlviii
Idiot Child, Help for an, xci, ciii
India, Medicine in, l
—— Nurses for, lvii
Infantile Mortality, xxxii
Infirmary Nursing, i, ix
—— Nurses and the Pension Fund, lxxxv
Influenza and Sisterhoods, lxix
Institutes, Nursing, i, vii, xxi, xxxiii, xlv, xlix, liii, lvii, lxxiii, lxxvii, lxxxi, lxxxix, xciii, xcvii, ci, cv
Intolerance at Canterbury Trained Nurses' Institute, xxxiii
Ipswich Nurses' Home, xxi

Jubilee Nurses' Institute, An Affiliated Branch of the, xxxiii

Kemerton Nursing Association, lxix

"Lancet" on the Popularisation of Nursing, The, xxxvii
Lay Nurses at Paris, Attack on the, lxix
Lawrence, H. Newman, on Medical Electricity, xcv
Lectures, Nursing, xxi, xlix, lvi, lxxviii, lxxxii, lxxxvi, xc, xciv, cii, cvi
Leith Hospital, Strike of Nurses at the, lxi
Lepers, Nursing the, lxv
Lewis, Dr. Percy G., on Nursing, lxxviii, lxxxii, lxxxvi, xc, cii, cvi
Liverpool, The Nurse Dolls at, lxix

Manchester District Work, xxxvii
Marriage and the Pension Fund, xli
Martin, Dr. J. W., on Nursing Questions and Answers, lxxxvii
Mary Adelaide Letter, vii
Masso-Therapeutics, xliii
Matrons, A Hint to, ci
—— and the Metropolitan Asylums Board, xxv
—— Cottage Hospital, xv, xviii, xxiii
Melbourne, Report of the Alfred Hospital at, vii
—— Nurses at, lxi
Mental Disorder After Confinement, Early Symptoms of, xxv
Metropolitan Asylums Board on Sisters' Dresses, The, xxxiii
Meum and Tuum, lxxxvii, xcix, cvii
Middlesbrough, A Nurses' Home for, xlix
Midwife and Monthly Nurse Registration, lix
Midwives' Institute, lxxvii
—— Incorporation of the, xli
—— Registration of, xv, xxix, xciii
Mildmay, The Beginning of, xxxix
Moore, Sir William, on Medicine in India, l
Murrell, Dr., on Masso-Therapeutics, xliii

Naval Nursing Sisters, lxxxix
Netley, Nursing at, xxxix
Newcastle, Nurses' Home at, liii
New Year's Gift, A, xi
Nightingale, The, lxxiii
North London Nursing Association, lxxxix
Northampton Institute, lvii
—— Lecture at, xlv
Nurse, Every Woman a, xxxvii
—— The Recollections of a, xcv
—— The Title of, lxxxiii, xcix
Nurses' Case Book, A, lvii, lxv
—— and Health, xxxvii
—— Temperance, xvii
—— Cottage, lxi
—— Holiday Home for, lxxvii
Nursing for Sixpence, lxxxvii
—— Lectures, xxi, xlix, lvi, xciv
—— by Dr. Percy Lewis—
Introductory, lxxviii
Chest Diseases, lxxxii
Symptoms in Chest Diseases, lxxxvi
The Care of Chest Diseases, xc
Chief Dangers of Chest Cases, cii
The Abdomen, cvi
—— Medical and Surgical, lxvi
—— Obstetric, lxxi, lxxiv
—— on the Continent, xxxvii
—— Popular, xcvii
—— Questions and Answers, lxxxvii

Obstetric Nursing, lxxi, lxxiv
Old Jeremy's Visitor, liv
Older Nurses and their Use, The, lxxvi
Operation Case, A First, lxxxvii
Opiates, Notes on, l, lviii
'Opkins at the Fifth Avenue Theatre, xxv
Overworked Nurses, xlvii, lvi, lxiv, lxviii
Oxford, Notes from, xxix

Pay, More Nurses or Higher, lxxiii
Pauper Nurses to be Abolished at Richmond, xlv
Pension Fund, The, xvii, xxv
—— Affiliation of the Torbay Hospital to the, lxxxix
—— Marriage, The, xli
—— Cottage Hospitals, The, lxix
—— its Enemy, The, lxxv
—— at the Mitchell Home, Torquay, cv
—— Honours for the First Thousand Nurses who Joined the, xcvii. cv
—— Increase of the Bonus Fund of the, lxix, lxxiii, lxxvii, lxxxi, lxxxv, lxxxix, xciii, xcvii, ci, cv
—— Insurance Journals on the, xxxviii, xlvii
—— Third Annual General Meeting of the, xcviii
Philadelphia News, lxi

Phlegmasia Dolens, Lecture on, xlix
Plaistow, Midwifery at, lxxix
—— Nursing Sisters at, xxv
Poetry—
Fair Dispensers, xliv
In Loving Memory, xviii
Our Nurse, xx
The Smallest Things, xlviii
Poor, Nurses for the, lxxxv
Probationers, Troublesome, xvii
Proxy at Brighton, Voting by, xxxix
Private Nurses, The Duties and Conduct of, ix

Registration and the British Nurses' Association, Voluntary, lxxx, lxxxi
—— Certificates and, li, lv
—— Monthly Nurse and Midwife, lix
—— of Midwives, xv, xxix, xciii
—— Refusal of the Hospitals to Take Part With the British Nurses' Association in Scheme of, lxix
Registry Office for Trained Nurses, A Co-operative, xxvii, xxxix, xliv, lxxxi
Reviews—
A Manual of Family Medicine and Hygiene for India, l
A Manual of Nursing, Medical and Surgical, lxvi
District Nursing, lxxxvii
Masso-Therapeutics, xliii
Minor Surgery and Bandaging, l
Nursing Questions and Answers, lxxxvii
Nursing for Sixpence, lxxxvii
Obstetric Nursing, lxxi, lxxiv
The Recollections of a Nurse, xcv
Toilers in London, xliii
Richardson, Dr., Criticism on, ix
Risks Which Nurses Should Run, The, xxvii
Rome, Secessions to, liii
Rural Nursing Association, liii, lxix, lxxvii

Salford Infirmary, Scandal at, xiii, xvii
Salisbury Infirmary Walk, i
Savage, Dr., on Mental Disorder after Confinement, xxv
Scarborough Nurses' Institute, xcvii
Selly Oak Workhouse, Scandal at, xxxvii, xli
Sermon, The Story of a, xciv
Sheffield Union, Nursing at the, lxxiii
Sickness, In Days of, lviii, lxxiv
Sisterhoods and Influenza, lxix
South Devon Hospital, lxxxix
—— Hants Infirmary, New Nursing Scheme for, lxv
Stalybridge, District Nursing at, liii
Stewart, Miss Henrietta, lvii
St. Kilda, Nurse at, i
Surgeons, The Two, xviii, xxii
Surgery and Bandaging, Minor, l
Superannuated at Forty-three, ix, xviii

Temperance, Nurses and, xvii
"They," The Bitter Cry of, viii
Thompson, Notes on Lectures by Dr. Symes, l, lviii
Toilers in London, xliii
Trantum's Hobby, Mr., lxvi, lxx

Uniforms, The Exhibition of Nursing, iii, xiii, xxix, xxxix, xlii, xlvi, lii, lvii, lxix, lxxix, xciii
—— Rights and Wrongs of Nursing, lxii
Union Infirmaries, Carelessness at, xxi
—— Nurse, The Duties of a, ciii
United States, A Letter from, lvii
University College, Notes from, xxi

Valeria, Miss, v
Village Nursing, vii, xxiii
Voting by Proxy at Brighton, xxxix

Wakefield, A Nursing Institute for, xlix
Ward Decoration, xxxviii
Warneford Hospital, Dispensing at the, ci
Warwick House, Leeds, xxix
Wigan Infirmary Nurses, xxi
Wilson, Miss, on Ladies and Workhouse Infirmaries, lxxv
Winchester, The Christmas Entertainment at the Royal Hants County Hospital, lxxi
—— Training School for Nurses, lxxviii
Wood, Miss, Lecture by, xlv
Worcester News, xciii
Workhouse Infirmaries, Ladies and, lxxv
—— Infirmary Nursing Association. Report of the, lxxx
—— Nursing Association, Miss Twining on the, lxxiii
Workhouses, Nursing in, viii

Zenana Medical College, The, ciii

### NOTICE TO CORRESPONDENTS.

All MS., Letters, Books for Review, and other matters intended for the Editor, should be addressed The Editor, The Lodge, Porchester Square, London, W.

### Notice to Advertisers and Subscribers.

All Advertisements, Orders for Copies of The Hospital, and Business Communications should be addressed to The Publisher, *not to the Editor*, The Hospital, 139-140, Salisbury-court, Fleet-street, E.C.

Vol. V., now ready, 4s., post free. Cases for binding, may be had of the Publisher, at 1s. 6d. each.

### To Residents, Secretaries, and Matrons.

The Editor will be glad to have the Regulations and each Report as issued by Asylums, Hospitals, Convalescent and Nursing Institutions, as well as those of other Charities, and an early copy in duplicate of all Appeals and Festival Notices, etc., addressed to *The Editor, The Lodge, Porchester-square, London, W.* Any superintendent, secretary, matron, or other chief official who regularly sends such information may be registered, on application to the Publisher, to receive free of cost a cover or binding The Hospital in half-yearly volumes.

### ALGERIAN HINTS FOR TOURISTS.*

### Scraps and Gleanings.

Diphtheria has appeared at Morden.

Hastings Hospital Saturday realised £257.

A horse has died in Yorkshire from hydrophobia.

The death is announced of Dr. Thomas Blunt, of Leicester.

A harvest thanksgiving service was held in Epsom Workhouse.

Ten per cent. of the Cairo garrison are in hospital with enteric fever

At Trepassy, Newfoundland, there is a severe epidemic of diphtheria.

Mr. D. F. Whiteley has been appointed house surgeon to Wallasey Dispensary.

Mr. Fox, dentist at North Shields, is using a new anæsthetic he calls vitalised air.

Warneford Hospital has received £247 from the local Hospital Saturday Fund.

Mr. J. Crisp has been appointed house surgeon and secretary to Stamford Infirmary.

St. Thomas's and the London Old Students held their respective annual dinners on October 1.

Mrs. Steward Ker has been appointed to the honorary medical staff of Wirrall Children's Hospital.

A painter has died at the London Hospital of hydrophobia. He was bitten by a rabid dog last May.

The Marchioness of Dufferin lately laid the foundation-stone of a convalescent home for children at Knockbreda.

Sir Henry Acland has visited Newcastle Infectious Hospital and expressed his approval of the arrangements.

An examination for seventeen appointments in Her Majesty's Indian Medical Service will be held next February.

The entrance to the Royal Victoria Hospital, Montreal, is to be like the famed doorway of Fyvie Castle, Aberdeenshire.

Dr. Norman Kerr took the chair at the first meeting for the season of the Society for the Study of Inebriety last Tuesday.

The Elephant Man has been for a holiday in the country, and has now returned to his winter quarters at the London Hospital.

The Rev. A. K. M. Wilshere, chaplain of Robben Island, has written his account of the lepers there and the treatment they receive.

The anniversary service of the Guild of St. Luke will be held at St. Paul's Cathedral on October 18th at 7 p.m. Preacher, Rev. T. B. Dover.

The Governor-General of Quebec and Lady Carron lately visited Beauport Lunatic Asylum and expressed their satisfaction with all they saw.

A very successful bazaar has been held at Huntley in aid of the Jubilee Cottage Hospital. The Duke of Richmond and Gordon was present.

The Agricultural Labourers' Union has had to reduce its scale of sick-pay, owing to the extraordinary amount of illness amongst the members last year.

Dr. Lander Brunton has been selected to go to Hyderabad to examine the experiments on the administration of chloroform there carried on.

The Loyal United Friends held a demonstration last Sunday in aid of the London Homœopathic Hospital. The Rev. Dacre Craven preached the sermon.

Whooping cough is very prevalent amongst the juvenile population of Wallington and the district, four deaths having been recorded during the last few days.

Edinburgh Royal College of Surgeons offers a prize of thirty-five guineas for the best essay sent in by October 26th by a fellow or licentiate of their college.

The East Anglian Branch of the British Medical Association held its half-yearly meeting at Braintree, under the presidency of Dr. J. Sinclair Holden, of Sudbury.

Dr. Carpenter has written to the Croydon Board of Guardians about the foolish statements the anti-vaccinationists lately made before Croydon Borough Bench.

A schoolgirl has died from hydrophobia at St. Thomas's Hospital, London. The girl's father and brother, who were bitten by the same dog, started for M. Pasteur's institute.

Sir Morrell Mackenzie has been presented with the freedom of the city of San Remo, richly illuminated and enclosed in an elegant casket, in recognition of his patronage of that city.

Like the workmen of Leeds, the artisans in the employ of the Wallsend Slipway Company have just completed the purchase of an ambulance carriage, built and equipped to their order by Messrs. Atkinson and Phillipson.

Says the *Sunday Times* about the proposed Protestant brotherhood: "It is everywhere admitted by the intelligent classes that poverty under all circumstances should necessarily involve celibacy. No religious obligations should be required to keep beggars out of matrimony."

The following announcement of the British Association caused much amusement: "Lady members and associates accustomed to dispense entirely with the use of the corset are requested kindly to present themselves at the Anthropometric Laboratory (Section H) particularly, in order that their breathing capacity may be gauged."

Mr. John Hewetson, of Brighton and Queen Victoria-street, whose residuary estate, subject to his wife's life interest, is to be held in trust in equal shares for the Refuge for Homeless Boys, the Asylum for Fatherless Children, the Aged Merchant Seamen's Institution, and the Royal Hospital for Incurables, leaves a personalty of £17,000.

### *SPECIAL NOTICE.*

*The Fiction and Amusements sections will be found in the* Nursing Supplement, *where they will appear for the future.*

